# Rheumatologist’s expertise in estimating risk of developing rheumatoid arthritis in patients with clinically suspect arthralgia: what is the value?

**DOI:** 10.1136/rmdopen-2025-006473

**Published:** 2026-02-20

**Authors:** Stijn Claassen, Hanna W van Steenbergen, Annette H M van der Helm-Van Mil

**Affiliations:** 1 Department of Rheumatology, Leiden University Medical Center, Leiden, The Netherlands; 2 Department of Rheumatology, Erasmus Medical Center, Rotterdam, The Netherlands

**Keywords:** Arthritis, Rheumatoid, Epidemiology, Sensitivity and Specificity

## Abstract

**Introduction:**

Clinical expertise is paramount in medical decision-making. In the setting of arthralgia, this expertise is highly relevant in differentiating clinically suspect arthralgia (CSA) from other musculoskeletal symptoms. However, it remains unclear whether rheumatological expertise is also reliable in estimating the risk of progression to rheumatoid arthritis (RA) in CSA patients. Using clinical expertise is more time-efficient than applying criteria, such as the European Alliance of Associations for Rheumatology/American College of Rheumatology (EULAR/ACR) risk stratification criteria for RA development. This study assessed the accuracy of RA-risk estimation based on rheumatologists’ clinical expertise and compared this to these criteria.

**Methods:**

501 CSA patients from the Leiden CSA cohort and placebo arm of the TREAT EARLIER trial were studied. At baseline, rheumatologists estimated RA risk (0–10 Numeric Rating Scale) informed by history, physical examination and laboratory results. The EULAR/ACR risk stratification criteria (without imaging) were calculated using baseline data. The outcome was RA development (2010 criteria) within 1 year, and the discrimination was compared.

**Results:**

Based on their expertise, rheumatologists estimated the risk of RA as a mean of 5 (SD 1.6) on a 0–10 scale. With an area under the curve (AUC) of 0.64 (95% CI 0.55 to 0.73), patients who did or did not develop RA were moderately differentiated. The RA risk was mainly overestimated compared with observed progression rates. In comparison, the EULAR/ACR risk stratification criteria in the same patients yielded an AUC of 0.91 (95% CI 0.87 to 0.95).

**Conclusions:**

Rheumatologists’ clinical expertise is inaccurate in assessing the risk of developing RA in patients with CSA, due to risk overestimation. This may support the use of established criteria when risk information has clinical or therapeutic implications.

**Trial registration number:**

NTR4853-trial-NL4599

**WHAT IS ALREADY KNOWN ON THIS TOPIC**

- Clinical expertise is essential in differentiating patients with clinically suspect arthralgia (CSA) from other musculoskeletal symptoms. Yet, within CSA patients, its accuracy in predicting the development of rheumatoid arthritis (RA) is unclear.

**WHAT THIS STUDY ADDS**

- The clinical expertise of rheumatologists moderately discriminates between CSA patients who do and do not progress to RA, as the clinical expertise tends to overestimate the risk.
- Risk quantification by the European Alliance of Associations for Rheumatology/American College of Rheumatology criteria for RA development demonstrated substantially higher predictive accuracy.

**HOW THIS STUDY MIGHT AFFECT RESEARCH, PRACTICE OR POLICY**

- Although clinical expertise is highly relevant in the diagnostic phase of arthralgia, it is insufficient for prognostication in patients with CSA.
- These findings highlight the limitations of relying solely on clinical judgement for RA risk and suggest that the use of validated risk stratification methodology is useful.

## Introduction

 Clinical expertise is paramount in medical decision-making. Although diagnostic and treatment recommendations are important, clinical expertise is crucial in making decisions in individual patient care. Its importance is also evident in research on rheumatoid arthritis (RA). For instance, the 2010 classification criteria for RA were developed using methotrexate use as a reference, which served as a proxy for rheumatological expertise in determining the diagnosis of RA.^1^ Similarly, the Disease Activity Score was developed with clinical expertise (expressed as treatment change) as a reference.^2^

In the context of arthralgia at risk for RA, clinical expertise has proven to be highly valuable as well, particularly in differentiating clinically suspect arthralgia (CSA) from other musculoskeletal symptoms.^3^ After identifying CSA, the next critical challenge is to distinguish among all CSA patients those who will develop RA from those who will not. Patients at high risk for RA could be prioritised for future trial eligibility and closer monitoring in clinical follow-up, while those at low risk may be reassured and spared unnecessary rheumatology follow-up at the outpatient clinic. To address this issue, the European Alliance of Associations for Rheumatology/American College of Rheumatology (EULAR/ACR) risk stratification criteria were developed to create a homogeneous population at risk for research purposes.^4^ Whether these are more discriminating than clinical expertise, or whether clinical expertise performs about as well, remains to be determined.

Clinical expertise might offer a more time-efficient approach to risk stratification. In contrast, using criteria, such as the EULAR/ACR criteria, requires the structured assessment and weighting of multiple characteristics, and this application involves tedious calculations. Clinical expertise, however, does not require a minimum set of variables, is estimated instantly (although honed through years of experience), and can take into account subtle contextual factors that standardised models did not capture. Therefore, this study investigates whether a 0–10 risk score, based on the rheumatologist’s clinical expertise, can offer similar prognostic accuracy for RA development as the EULAR/ACR risk stratification criteria, based on clinical and serological variables.

## Methods

### Patients and study design

We studied clinically suspect arthralgia patients who were consecutively included in the observational Leiden CSA cohort or the placebo arm of the TREAT EARLIER trial at the Leiden University Medical Center (LUMC); both the cohort and trial are described elsewhere.^5 6^ In short, in this cohort and trial, adults with recent-onset (<1 year) arthralgia of the small joints were included, who were deemed at risk for RA by their treating rheumatologist. By definition, patients with clinical arthritis at physical exam or a more likely other cause of joint pain (eg, osteoarthritis or fibromyalgia) were excluded.

### Risk assessment based on clinical expertise

In both settings, the baseline visit included a risk assessment for RA development based on the clinical expertise of the treating rheumatologists. For the present analysis, patients enrolled between 2015 and 2021 were studied from the CSA Leiden cohort; 2015 marked the start of recording the rheumatologist’s risk estimation. Enrolment for the TREAT EARLIER trial occurred between 2015 and 2019. All TREAT EARLIER patients were seen at the outpatient clinic of the LUMC. Although 2012 marked the start of the CSA cohort, it was only after 3 years of experience with this patient population that we began recording the rheumatologist’s risk estimation, ensuring the risk assessment was performed by clinicians who gained sufficient expertise to provide reliable and consistent evaluations.

During the specified inclusion periods, a team of 9 rheumatologists and up to 33 rheumatology fellows at the rheumatology outpatient clinic of the LUMC were involved for both the CSA cohort and the TREAT EARLIER trial. The rheumatology fellows are physicians in training to become rheumatologists and have at least 3 years of clinical experience. All clinical evaluations at the outpatient clinic are reviewed on a case-by-case basis by the supervising rheumatologist, ensuring specialist oversight.

Risk estimation was performed after a comprehensive medical history, physical examination, and, in nearly all cases, review of standardised laboratory tests, including IgG anti-citrullinated peptide antibody (ACPA), IgM rheumatoid factor (RF) and C reactive protein (CRP). Based on their clinical expertise, they estimated the patient’s likelihood of developing RA on a Numeric Rating Scale ranging from 0 to 10 (from low to high predicted risk of RA). MRI findings were obtained afterwards and were not disclosed to the rheumatologists at the time of risk estimation.

### Application of EULAR/ACR risk stratification criteria

For comparison, the risk of progression to RA was also quantified using the EULAR/ACR risk stratification score, based on clinical and serological variables (range: 0–26).^4^ This risk assessment includes morning stiffness duration, patient-reported joint swelling, difficulty making a fist, increased CRP, RF level and ACPA level. Imaging findings were not incorporated to facilitate the best comparison with the rheumatologist’s clinical expertise. There is a partial overlap (approximately 22%) between the current study population and the derivation dataset of the EULAR/ACR risk stratification criteria.

### Outcome

Patients were prospectively studied; follow-up data for up to 1 year were studied. The primary outcome was defined as the development of RA, which was characterised by clinically apparent inflammatory arthritis and fulfilment of the 2010 ACR/EULAR criteria within 1 year.^1^ During a follow-up, participants were not allowed to take disease-modifying antirheumatic drugs.

### Statistical analysis

To test the discrimination between RA progressors and non-progressors after 1 year, we calculated the area under the receiver operating characteristic (ROC) curve (AUC) for both scores (rheumatologist’s clinical expertise and EULAR/ACR risk stratification score) and compared them using DeLong’s test. Across the individual variables included in the EULAR/ACR risk stratification model, missingness was low (ranging from <1% to 12% per variable), with 79% being complete cases. Missing values of variables necessary for the EULAR/ACR risk stratification were imputed using multiple imputation using chained equations. Furthermore, to test how well the predicted scores by the rheumatologist correlate with the observed risk, we plotted a calibration curve showing the observed risk versus the predicted risk by the rheumatologist. Further details on missingness are provided in online supplemental file 1. A complete case analysis was also added. In a sensitivity analysis, we assessed whether the discriminative ability of the clinical risk estimates was consistent across rheumatologists and rheumatology fellows. ROC curves were generated separately for each group, and AUCs were compared using an independent-sample test based on the χ² statistic, as the two groups evaluated different patients. Lastly, we tested the discriminative performance of the clinical expertise risk for RA progression score within strata of ACPA status.

Statistical analyses were performed using SPSS V.19 and Stata V.16.1.

## Results

### Clinical expertise and evaluations

A total of 501 patients were studied: 387 from the CSA cohort and 114 patients from the TREAT EARLIER placebo arm. Their baseline characteristics are listed in table 1. At baseline, 501 evaluations were completed by 42 clinicians (9 rheumatologists, 33 fellows). Of these, 140 (28%) were performed by a rheumatologist directly and 361 (72%) by rheumatology fellows under their supervision. The number of evaluations per clinician ranged from 1 to 42, with a median of 9 (IQR 4–18), indicating a balanced distribution of assessments across the team.

**Table 1:** Baseline characteristics

Characteristic	All patients (n=501)
Age in years	44.1±12.1
Female	381 (76%)
First-degree relative with RA	136 (28%)
Symptom duration in days	103 (38–252)
Morning stiffness >60 min	173 (39%)
Tender joint count-68	5 (2–10)
CRP elevated (>5 mg/L)	109 (22%)
RF positive (>3.5 IU/mL)	104 (21%)
ACPA positive (>7 U/L)	75 (15%)
Total MRI-inflammation (RAMRIS)	2.5 (1.0–5.5)

Continuous variables are presented as means±SD, binary variables as number (percentage) and non-normally distributed continuous variables as median (IQR).

ACPA, anti-citrullinated protein antibodies; CRP, C reactive protein; RA, rheumatoid arthritis; RAMRIS, Rheumatoid Arthritis Magnetic Resonance Imaging Score; RF, rheumatoid factor.

**Figure 1:**
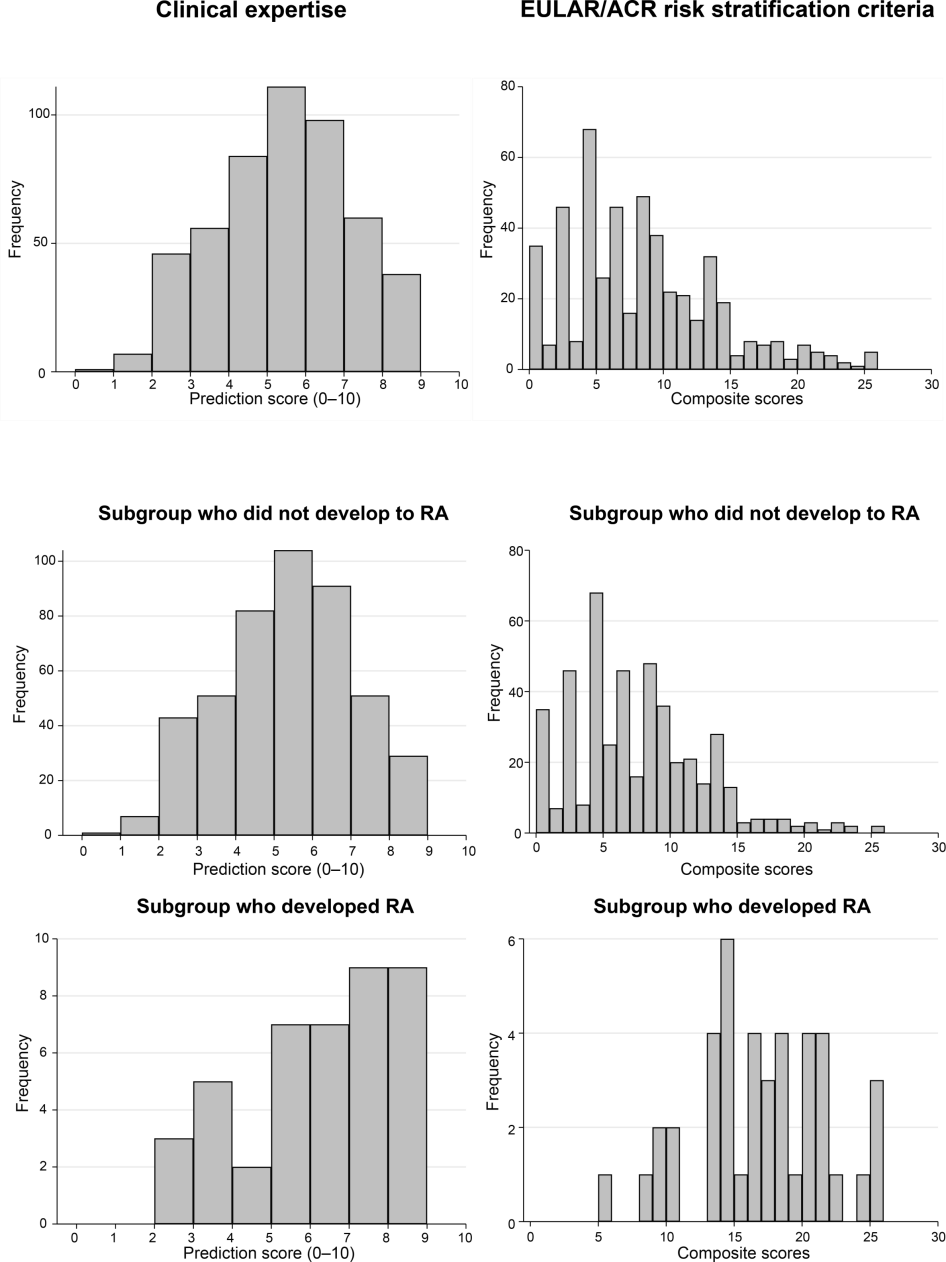
Distribution of prediction scores of the clinical expertise (assessed on a 0–10 scale; left column) or the RA risk scores of the EULAR/ACR risk stratification criteria for RA development (right column) in the total patient group, and split for the patients who did and did not progress to RA in the first year. EULAR/ACR, European Alliance of Associations for Rheumatology/American College of Rheumatology; RA, rheumatoid arthritis.

**Figure 2:**
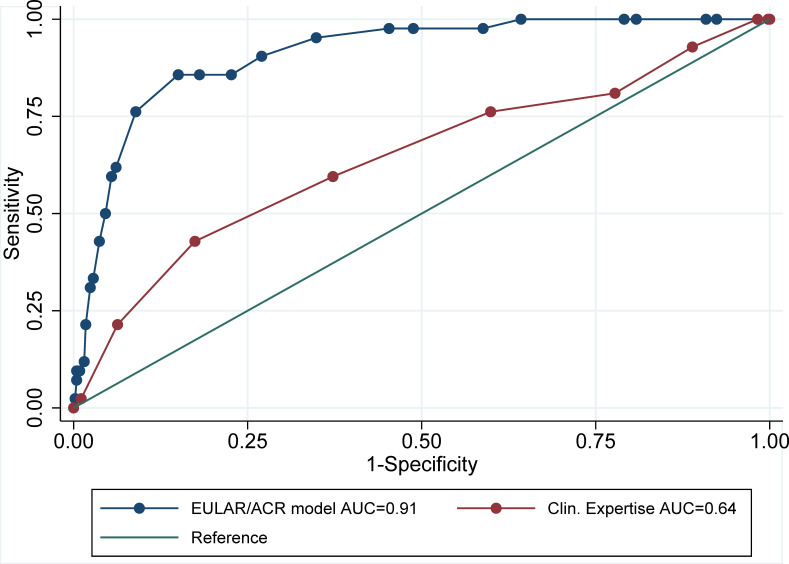
Receiver operating characteristic curve for prediction of progression to RA within 1 year, comparing the clinical expertise-based risk prediction score and EULAR/ACR risk stratification criteria based on clinical and serological variables. AUC, area under the curve; EULAR/ACR, European Alliance of Associations for Rheumatology/American College of Rheumatology; RA, rheumatoid arthritis.

The mean baseline risk score for RA development, based on clinical expertise, was 5.0 (SD 1.8), with the observed scores following a normal distribution. Most patients had a risk score between 4 and 6 on a 0–10 scale (figure 1). The rheumatologist’s risk prediction score had an AUC of 0.64 (95% CI 0.55 to 0.74) for predicting RA development in CSA patients at 1 year (figure 2). When comparing the rheumatologist risk scores in progressors and non-progressors, a slight increase in score is visible in the patients who progressed to RA (figure 1).

**Figure 3:**
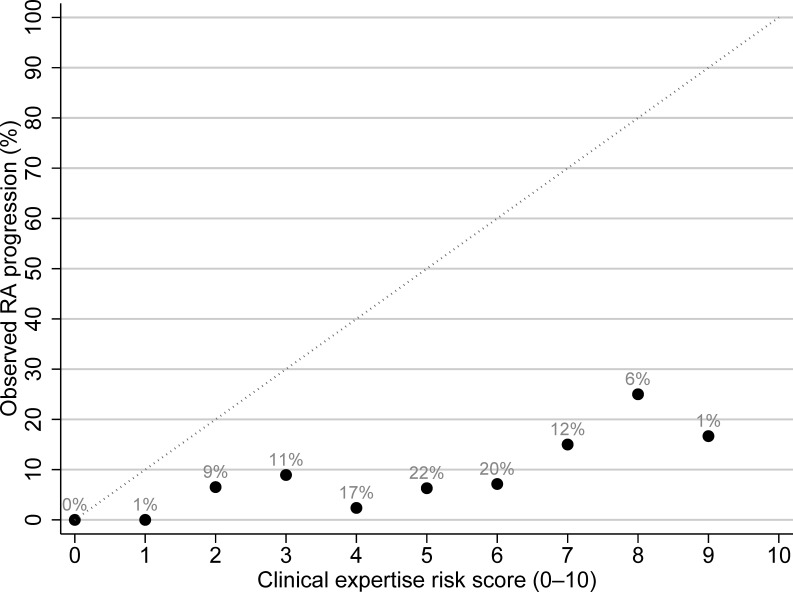
Calibration plot showing observed progression to RA within 1 year plotted by the rheumatologist’s risk prediction score at baseline. The grey dotted diagonal line represents perfect calibration, where estimated and observed RA progression rates align. For example, a score of 1 would correspond to a 10% observed RA progression at 1 year. The black dots show the actual calibration, and the numbers above the dots indicate the proportion of patients with this score as a percentage of the total (n=501). RA, rheumatoid arthritis.

When plotting the observed risk against the estimated risk based on clinical expertise, an overall overestimation of the risk of RA development is evident (figure 3). For instance, in patients assigned a risk score of 7 (on a 0–10 scale), an observed risk of 70% would have been expected, yet only 20% of patients progressed to RA. Similarly, less than 30% of patients with a score of 8 (on a 0–10 scale) developed RA.

### Sensitivity analysis

Comparable discriminative performance in RA development was observed between rheumatologists and fellows. The AUC was 0.65 (95% CI 0.41 to 0.87) for rheumatologist versus 0.65 (95% CI 0.54 to 0.75) for fellows; p=0.98 for equality of AUCs (independent sample ROC comparison). Discrimination performance dropped when stratified for ACPA status (AUC=0.51 in both ACPA-positive and ACPA-negative subgroups), likely reflecting reduced heterogeneity within strata.

### EULAR/ACR risk stratification score

Applying the EULAR/ACR risk stratification score, which uses clinical and serological variables, in the same patient group yielded an AUC of 0.91 (95% CI 0.87 to 0.95). This is significantly higher than the AUC of the clinical expertise-based score (p<0.001). The complete case analysis (n=396) yielded a similar AUC of 0.90 (95% CI 0.85 to 0.95). When comparing the scores of the patients who progressed to RA within 1 year to those who did not, a clear separation is visible (figure 1). The distinction between progressors and non-progressors is clearer in comparison to the rheumatologist risk score.

## Discussion

In this study, we assessed the predictive value of the rheumatologist’s clinical expertise for RA development in patients with CSA to determine whether clinical expertise alone is sufficiently accurate for prognosis. We established that this expertise could not adequately discriminate between CSA patients who did and did not develop RA within a year.^3^ Plotting predicted versus observed risk showed that clinicians generally overestimated the risk of RA. In contrast, within the same patients, the EULAR/ACR risk stratification criteria demonstrated substantially better discriminatory performance.

Previously, clinical expertise has been proven highly effective when distinguishing CSA from other types of arthralgia (OR 55, sensitivity 93%).^3^ Adding the EULAR CSA definition criteria for diagnosis on top of that had a relatively minor added value for the diagnosis of CSA (HR 2.1).^7^ However, the current data revealed that determining a prognosis (ie, estimating the risk of RA development) within CSA patients proved far more challenging based solely on clinical expertise. This distinction is important, since the risk of RA development lies at the heart of the clinical management of patients with CSA, when selecting patients for a new prevention trial or when determining the intensity of follow-up in daily clinical practice. The difference in accuracy may lie in the nature of the task: cross-sectional recognition of signs and symptoms as part of diagnosing an at-risk state is perhaps inherently easier than predicting long-term outcomes. The field of oncology offers a parallel: while clinical expertise remains crucial for diagnosing cancer and providing care, prognosis relies heavily on objective tools such as the tumour-node-metastasis staging system or genetic markers (eg, BRCA mutations in breast cancer), which stratify patients by risk of recurrence or survival, and thereby guide treatment decisions and follow-up intensity.^8^

Clinical expertise is an inherently abstract concept; despite standardised medical training, individual experiences and expertise of the rheumatologist may influence how the risk of RA development is assessed. This variability makes it challenging to determine which patient characteristics contribute most to the risk estimation and whether they are weighed in a similar manner. Although we could not provide an analysis on which factors clinicians considered most important for assessing the risk according to their expertise, since we did not specifically ask the clinicians, the data did reveal an overall lack of accuracy, as rheumatologists tended to overestimate the risk of RA. Conceptually, overestimating RA risk in the at-risk stage of arthralgia can lead to overmedicalisation or overcautious follow-up strategies, both resulting in increasing burden on patients and the healthcare system without clear benefit. In this context, the time investment of using validated risk stratification criteria when deciding on clinical management or trial inclusion seems worthwhile.

Furthermore, risk prediction tools and classification criteria can also influence clinical expertise and management over time. For example, the high weighting of autoantibodies (ACPA and RF) in the 2010 EULAR/ACR RA classification criteria has also increased their value in the clinical diagnosis of RA and may even have fuelled discussions about the diagnosis of RA in the absence of autoantibodies, which were less prominent before the development of the 2010 criteria. Likewise, the remaining group of undifferentiated arthritis has changed since the 2010 classification criteria were introduced.^9^ Another example is the FRAX tool (a fracture risk calculator), whose integration into guidelines has not only improved hip fracture risk targeting and treatment uptake but also provided clinicians with probability estimates of fracture, creating an educational feedback loop.^10^ The EULAR/ACR risk stratification for the development of RA was designed for use in patients with joint pain suspected of progression to RA and is therefore used in addition to clinical expertise. These criteria may also influence the expertise-based risk estimates over time; this is a question for further research that can be conducted in a few years. Finally, it is important to note that the EULAR/ACR risk stratification criteria (also called classification criteria) were primarily developed for research purposes and not for individual patients in routine clinical practice.

A limitation of this study is that the performance (AUC) of the EULAR/ACR criteria may be slightly inflated, as part of our data used here was also used for the derivation of the EULAR/ACR criteria. However, this overlap is partial, as our data comprised only 22% of the data used to build the EULAR/ACR criteria, and internal validation using bootstrapping demonstrated robust AUC estimates, suggesting that the performance metrics are likely reliable even when applied to the derivation cohort. The partial overlap also explains why the AUC of the criteria reported here is slightly different from that reported for the derivation and validation phases of the criteria.^4^

It should be noted that the EULAR/ACR risk stratification criteria can be used without and with information on MRI variables (tenosynovitis). We used the criteria without MRI variables, as the clinicians lacked MRI data when making their predictions, making such comparisons inappropriate.

Another limitation is that the clinical expertise data originate from a single medical centre. Nonetheless, the sample of 42 experts (9 rheumatologists and 33 rheumatology fellows) should be sufficient to prevent the dependency on a single or a few experts. Importantly, no individual clinician contributed more than 42 scores (<10% of measurements), ensuring a broad representation. Although 72% of the evaluations were conducted by rheumatology fellows (medical doctors in training to become rheumatologists with 3–6 years of clinical experience), this has unlikely influenced results since all cases in the outpatient clinic are reviewed and supervised by a rheumatologist. Accordingly, rheumatology fellows and rheumatologists demonstrated comparable performance in this study. Furthermore, this group has previously demonstrated strong ability in recognising CSA, supporting the validity or quality of their clinical expertise in another task.^3^

In conclusion, rheumatologists’ clinical expertise seems insufficient in assessing the risk of developing RA in patients with CSA, due to an overestimation of risk. This may support the use of established criteria, such as the EULAR/ACR risk stratification criteria, when risk information has clinical or therapeutic implications.

## Supplementary material

10.1136/rmdopen-2025-006473 online supplemental file 1

## Data Availability

Data are available on reasonable request.
