# Supplementary material for: Rheumatologist’s expertise in estimating risk of developing rheumatoid arthritis in patients with clinically suspect arthralgia: what is the value?
Source: RMD Open. 2026 Feb 20;12(1):e006473. doi: 10.1136/rmdopen-2025-006473 (PMC12927288; doi:10.1136/rmdopen-2025-006473)
Supplement: online supplemental file 1 [file rmdopen-12-1-s001.docx]

# Supplementary file 1. Details on missingness

Overall, missing data were rare; among 501 patients, only 105 (21%) had any missing data on the six items necessary to calculate the EULAR/ACR risk stratification score.

Missingness rarely co-occurred: in only seven patients, two items were missing, and in one patient, three items were missing; otherwise, only one of six items was missing (supplementary table 1 and 2). We used Multiple Imputation by Chained Equations (MICE) to impute missing values for the components, and then recalculated the full risk stratification score by combining the imputed and observed values.

In nearly all cases with a missing EULAR/ACR risk stratification score, five components were observed, and only one was imputed, thereby minimising the impact of imputation on the total risk score.

We ran the MICE algorithm in STATA v16.1 with 40 imputations and a burn-in of 100 iterations. Binary variables were imputed using logistic regression and continuous variables using predictive mean matching. Convergence was monitored via trace plots, and post-imputation diagnostics were performed to ensure stability and adequacy of imputations.

The imputation model included all variables from the analysis model to preserve associations:
Increased CRP, 68 tender joint count, total HAQ-DI score, morning stiffness duration, patient reported swollen joint, rheumatoid factor level, ACPA level, symptom duration, age at inclusion, sex, difficulty making a fist, MRI-detected tenosynovitis extensor MCP, MRI-detected tenosynovitis extensors MTP, MRI-detected tenosynovitis flexors wrist, MRI-detected tenosynovitis extensors wrist, and EULAR/ACR scoring model.

**Supplementary Table 1.** Missingness table

| Item | N missing | Percentage missing |
| --- | --- | --- |
| Morning stiffness score | 58 | 11.6 % |
| Patient reported swollen joint score | 34 | 6.8 % |
| Difficulty making a fist score | 11 | 2.2 % |
| Increased CRP | 6 | 1.2 % |
| Rheumatoid factor level | 4 | 0.8 % |
| ACPA level | 1 | 0.2 % |
| Composite score of clinical and serological EULAR/ACR risk stratification (missingness of at least one of the variables to compute the model) | 105 | 21.0% |

**Supplementary Table 2.** Missing-value patterns (green means complete)

| Frequency | ACPA level | RF level | Increased CRP | Difficulty making a fist | PT swollen joint count | Morning stiffness |
| --- | --- | --- | --- | --- | --- | --- |
| **396** |  |  |  |  |  |  |
| **51** |  |  |  |  |  |  |
| **29** |  |  |  |  |  |  |
| **10** |  |  |  |  |  |  |
| **5** |  |  |  |  |  |  |
| **4** |  |  |  |  |  |  |
| **3** |  |  |  |  |  |  |
| **1** |  |  |  |  |  |  |
| **1** |  |  |  |  |  |  |
| **1** |  |  |  |  |  |  |
